# Deformation and Constitutive Behaviors of Ferrite Steel Under Tension Condition

**DOI:** 10.3390/ma19091889

**Published:** 2026-05-03

**Authors:** Hui Lin, Lin Lv, Xueli Ge, Tao Jin

**Affiliations:** 1 Geely Automobile Institute, Hangzhou Polytechnic University, Hangzhou 310018, China; linhuizipc@163.com; 2 Hangzhou Geely Automobile Co., Ltd., Hangzhou 310020, China; gexueli@geely.com; 3 School of Intelligent Manufacturing, Taizhou University, Taizhou 318000, China

**Keywords:** ferrite steel, anisotropic constitutive behaviors, associated flow rule, microstructures, fracture failure

## Abstract

This study systematically investigates the tensile anisotropic mechanical behavior of ferritic steel under different orientations through an integrated experimental, theoretical modeling, and simulation approach employing advanced characterization techniques including electron backscatter diffraction (EBSD), digital image correlation (DIC), scanning electron microscopy (SEM), and finite element analysis. The results demonstrate pronounced orientation dependence in mechanical response, with initial yield strengths of 391, 391, and 405 MPa and fracture strains of 0.237, 0.220, and 0.212 observed for 0°, 45°, and 90° orientations, respectively, corresponding to orientation-induced variations of 3.6% in yield strength and 11.8% in fracture strain. These anisotropic characteristics are primarily attributed to the predominant α-fiber texture <110>||RD, which accounts for 59.8% of the texture components. Furthermore, crystallographic texture significantly influences fracture behavior, as evidenced by the distinct orientation-dependent macroscopic contraction characteristics and morphological features of fracture surfaces.

## 1. Introduction

Ferritic steels are widely utilized in automotive, construction, and energy sectors due to their high strength, corrosion resistance, and excellent manufacturability [1]. In architectural applications, their superior durability, recyclability, and corrosion resistance eliminate the need for toxic protective coatings, positioning them as sustainable building materials suitable for load-bearing structural components [2,3]. Within the automotive industry, ferritic steels are predominantly employed for structural parts, fasteners, and fuel tanks. Consequently, from a safety perspective, the mechanical properties of ferritic steels represent one of their most critical characteristics [4]. Furthermore, a comprehensive understanding of their mechanical behavior is of significant value, whether for optimizing forming processes or evaluating in-service reliability [5].

Existing research on ferritic steels has been extensively conducted [6,7,8]. Numerous studies have focused on the effects of forming processes, i.e., rolling reduction and annealing temperature on the microstructural evolution and mechanical properties of ferritic steels [9,10,11,12]. Zhao et al. found that the rolling schedule with a first pass reduction of 40% was optimal for reducing the proportion of undesired texture components, such as {001}[110] and {114}[110] components, and this operation resulted in a maximum reduction of 40% in the ridging height of FSS 430 foils. In addition, the annealing temperature of 900 °C was found to be effective in enhancing microstructure homogeneity, reducing dislocation density and improving the ridging resistance of ferritic steel foils [9]. Shukla et al. investigated cryogenic cooling and forging on the microstructure, mechanical, and corrosion behavior of AISI 430 ferritic stainless steel and found that cryogenic quenching led to a favorable strength–ductility balance, whereas cryogenic quenching with forging led to substantially increased yield strength at the expense of ductility [13].

Many studies focused on the relationship between microstructure evolution and the mechanical properties of metals [14,15,16,17,18]. The study by Aghamohammadi and Jamaati revealed that when the rolling reduction reached 60%, highly elongated ferrite grains and shear bands developed in the microstructure of AISI 430 ferritic stainless steel. With increasing strain, a shear texture initially formed, followed by the gradual strengthening of a deformation texture. Concurrently, the dislocation density rose with accumulated strain, resulting in the significant enhancement of both macro- and micro-hardness [6,19]. Chung et al., using a CP-FE model, studied the mechanistic relationship between the ridging and the γ-fiber texture of shear dominant deformation and found that severe shear strain-induced texture components are the major cause of ridging [20]. Lin et al. [21] studied the IR-stimulated hardening in F321 stainless steel through both experimental methods and numerical simulations and found that the stainless steel undergoes microstructural alterations, such as the formation of IR-stimulated defects (e.g., stacking fault tetrahedrons (SFTs), dislocation loops (DLs), and clusters), resulting in IR hardening. Additionally, several studies have focused on enhancing the properties of ferritic steels through the incorporation of various secondary phases [22,23,24]. In a further study, the ductility and toughness of ferritic stainless steel was found to be remarkably enhanced with Ce refiner [7].

Although significant progress has been made in ferritic steel research, limited studies have addressed the texture-induced anisotropic mechanical behavior of these materials. Given their extensive industrial applications, a comprehensive understanding of orientation-dependent mechanical properties and constitutive modeling holds substantial engineering significance [25,26]. Accordingly, this study investigates the following aspects: (1) the microstructural characterization of as-received ferritic steel to elucidate its microstructural features and texture components; (2) the tensile testing of specimens with different orientations, analyzing orientation effects on tensile behavior through load–displacement measurements; (3) the development of a constitutive model based on Hill48 criterion with associated flow rule, implemented in Abaqus/CAE via VUMAT user subroutine, incorporating a return mapping algorithm; (4) fractographic analysis using SEM to examine orientation-dependent fracture mechanisms.

## 2. Experimental Methods

The specimens investigated in this work were prepared from 1 mm thick ferritic steel sheets, which were supplied by Southern Steel Bar Trading Co., Ltd. (Guangzhou, China). To investigate orientation-dependent mechanical behavior, electron backscatter diffraction (EBSD) was first employed to characterize the material’s microstructural features, including grain size, phase distribution, micro-orientation patterns, and texture characteristics of the as-received material [3,17,27]. Additionally, standard tensile specimens were extracted from the initial plate using electrical discharge wire cutting, with specimens oriented at 0°, 45°, and 90° relative to the rolling direction to account for orientation effects. Mechanical testing was conducted using a universal testing machine (CMT5105A, SANS, Shenzhen, China) equipped with a 100 kN load cell with a loading rate of 1.5 mm/min, while deformation measurements were performed through the digital image correlation (DIC) technique. To facilitate non-contact strain measurement, all specimens were prepared with discrete, random speckle patterns on their surfaces prior to testing [3,28]. Fractographic analysis was carried out using scanning electron microscopy (Carl Zeiss AG, Oberkochen, Germany) to examine the fracture surface morphology and investigate the orientation-dependent fracture behavior. Fracture morphology was acquired using an accelerating voltage of 10 kV, with the vacuum set to High Vacuum mode at a pressure of approximately 1 × 10^−4^ Pa. In addition, the analysis of the grain-scale microstructure of the specimen was performed using a JEOL JSM-7100F field-emission scanning electron microscope (JEOL Ltd., Tokyo, Japan) fitted with an EBSD detector (HKL NordlysNano, Oxford Instruments NanoAnalysis, High Wycombe, United Kingdom). EBSD data processing was carried out with HKL Channel 5 and AZtecCrystal software version 2.1.

## 3. Result and Discussion

### 3.1. Microstructures

**Figure 1:**
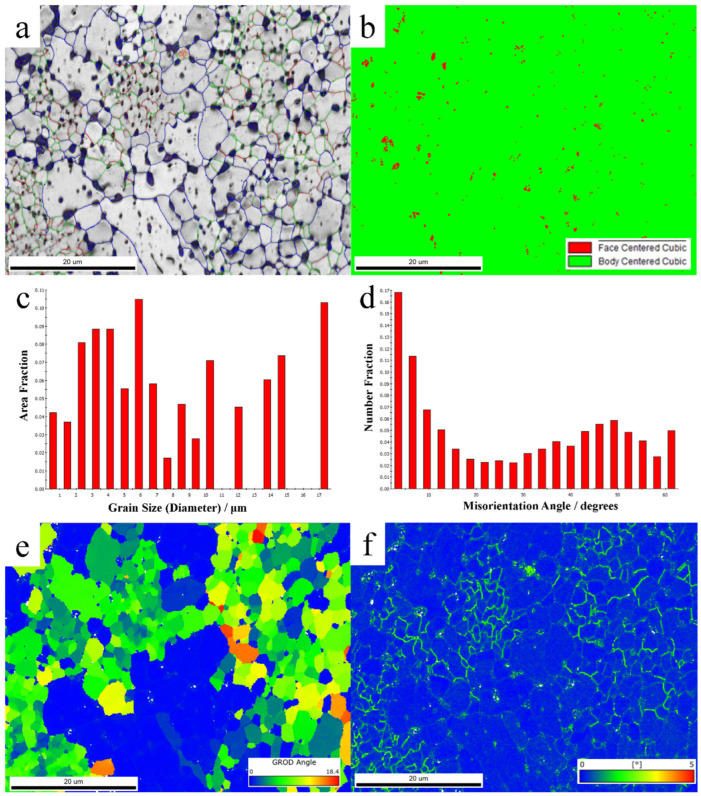
Micro-structures of the rolling ferrite steel before mechanical testing experiment. (**a**) EBSD-IQ map showing the representative microstructures; (**b**) phase distribution map; (**c**) grain size distribution in area fraction determined from the EBSD maps; inverse pole figures of three axes derived from pole figures measured with neutron radiation. (**d**) The local misorientation distribution map; (**e**) GROD images of sample; (**f**) KAM of ferrite steel sample.

Figure 1 presents the EBSD characterization results of the tested specimen, with a statistically determined grain count of 343. Figure 1a illustrates the microstructural characteristics of the specimen, revealing an average grain size of approximately 8.01 μm, with the size distribution depicted in Figure 1c. The proportion of subgrain boundaries (2–5°) is 0.166, primarily associated with dislocation walls formed by edge dislocations. Low-angle grain boundaries (5–15°) account for 0.241, mainly attributed to substructures such as dislocation cell evolution products or partial recrystallization. High-angle grain boundaries (15–180°) dominate with a proportion of 0.590, linked to recrystallization. Additionally, the specimen consists almost entirely of BCC ferrite phase, accounting for approximately 99.1%, as detailed in Figure 1b. Figure 1d displays the local misorientation angle distribution, exhibiting a bimodal pattern at both low and high angles, with the highest proportion at low angles, indicating the coexistence of extensive annealing recrystallization and substructures (e.g., subgrains and dynamic recrystallization) induced by severe deformation (rolling). To further investigate the presence of various microstructures, Figure 1e presents the Grain Reference Orientation Deviation (GROD) distribution. The fraction of GROD < 1° is 35.9%, suggesting the existence of recrystallized grains with a corresponding volume fraction. The range 1° < GROD < 5° (23.9%) corresponds to substructures such as dislocation cells, low-angle grain boundaries, and partial recrystallization, while GROD > 5° (40.2%) represents deformed grains with high dislocation density, pronounced orientation gradients, and elevated stored energy, which are prone to act as nucleation sites for recrystallization. For further clarification, Figure 1f provides the Kernel Average Misorientation (KAM) map of the scanned region. A comparison between GROD and KAM distributions reveals that regions with high GROD values coincide with those exhibiting high KAM values, confirming the simultaneous presence of recrystallized grains, grain substructures, and deformed grains in the specimen. This observation implies the potential existence of significant preferred orientation, leading to anisotropic mechanical behavior.

**Figure 2:**
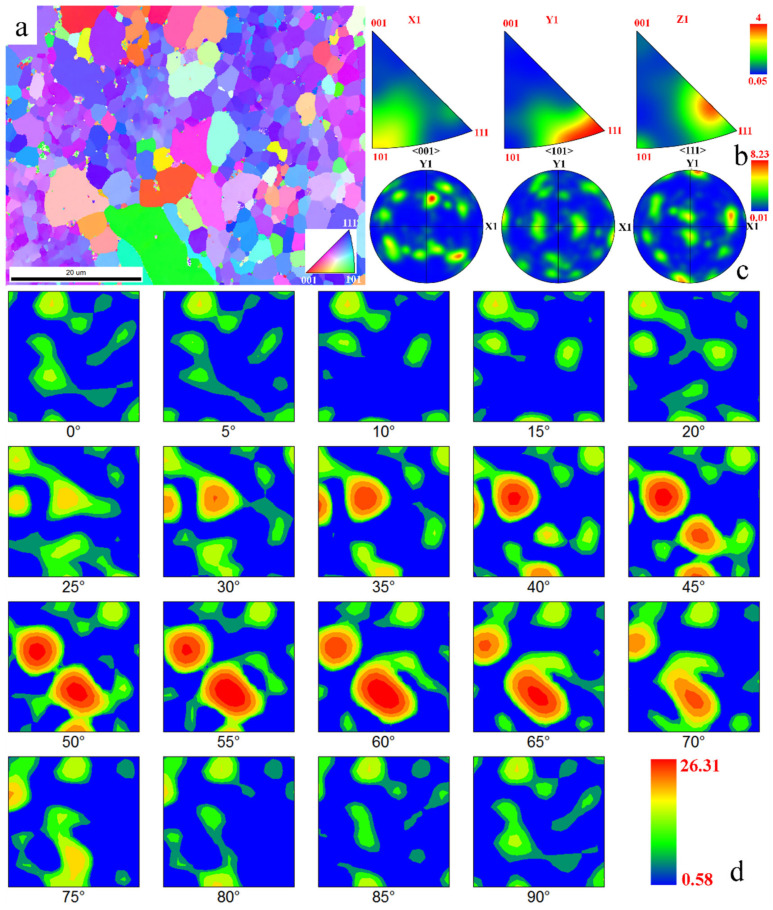
EBSD-IPF map showing the representative microstructures (**a**). Inverse pole figures of three axes derived from pole figures measured with neutron radiation (**b**); pole figures of the sample (**c**); ODF images of the sample (**d**).

Figure 2 presents the inverse pole images (EBSD-IPFx), IPF maps, and pole images of the primary phases in the specimen. As shown in Figure 2a, the grain orientations in the scanned region exhibit limited randomness, with a discernible <111>||RD texture. Additionally, an analysis of Figure 2b–d confirms the presence of {111}<112>, <110>||RD, <001>||RD, and brass textures. The {111}<112> texture, a γ-fiber texture, typically forms during recrystallization after cold rolling and annealing. The <110>||RD texture, an α-fiber texture, usually arises from partial recrystallization post cold rolling. The <001>||RD cubic texture is commonly associated with recrystallization annealing, while the brass texture, a deformation texture induced by plane strain during cold rolling, is a primary contributor to material plastic anisotropy. The <111>||RD texture also predominantly originates from cold rolling, primarily due to the activation of {110} and {112} slip systems, which promote <111> alignment along the rolling direction (RD). For the ferritic steel studied here, different textures exert varying influences on mechanical properties, necessitating further analysis of the distribution of distinct texture components.

**Figure 3:**
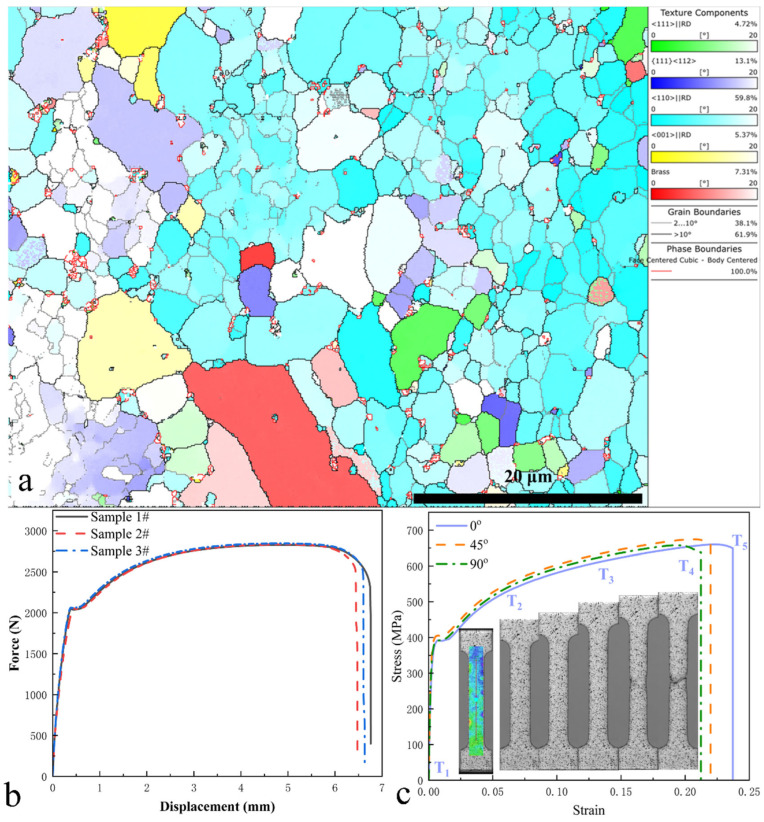
Texture distribution map (**a**) of rolling ferrite steel. Repeatable test results of force–displacement curves of different tested samples (**b**). Tension stress–strain curves of samples at different orientations of 0°, 45°, and 90° with illustrations of deformed sample at various loading stages (**c**).

Therefore, Figure 3a presents the texture distribution of the ferritic steel, dominated by the α-fiber texture <110>||RD (accounting for 59.8%), while the γ-fiber texture {111}<112> constitutes 13.1%, which is beneficial for deep-drawing formability. Additionally, minor components include <111>||RD (4.72%), <001>||RD (5.37%), and the brass texture {110}<112> (7.31%). These results demonstrate that the cold-rolled ferritic steel exhibits significant preferential orientation at the mesoscale, inevitably leading to anisotropic macroscopic mechanical properties. Consequently, it is necessary to investigate the mechanical behavior of specimens with different orientations, and the corresponding constitutive model must account for anisotropic characteristics. In addition, the strong <110>||D texture (≈59.8%) indicates that the local coordinate system of most grains is rotated relative to the global RD-TD-ND coordinate system by approximately 45° within the RD-TD plane. Under this texture condition, the three tested orientations correspond to specific local crystallographic directions: 0° (RD) corresponds to the [101] direction in the local grain coordinate system, 45° corresponds to the [010] direction, and 90° corresponds to the [210] direction. For a cubic crystal system, these three orientations effectively sample the primary mechanical responses of grains governed by the <110>||D texture. Therefore, testing at 0°, 45°, and 90° is considered sufficient to capture the orientation-dependent mechanical behavior of such material.

### 3.2. Anisotropic Stress–Strain Relations and Constitutive Modeling

Accordingly, Figure 3b presents the tensile load–displacement curves of different ferritic steel specimens, demonstrating good repeatability in the tensile data. Mechanical tests were subsequently conducted along three orientations relative to the rolling direction: 0°, 45°, and 90°. By integrating the load data from the testing machine and strain measurements obtained via digital image correlation (DIC), the stress–strain curves for the ferritic steel tensile specimens along these orientations were derived, as shown in Figure 3c. It should be noted that the strain values were determined using DIC technology by calculating the average strain over the virtual gauge length (illustrated in the inset of Figure 3c), employing the true strain formulation, while the corresponding stress was computed by dividing the load cell data by the initial cross-sectional area of the specimen and applying the true stress conversion. The stress–strain data for specimens with different orientations (right panel) reveal pronounced plastic anisotropy in the material. Notably, while the elastic response remains orientation-independent, the plastic regime exhibits significant orientation dependence in terms of initial yield strength, strain hardening behavior, and fracture characteristics. Furthermore, Figure 3c includes deformation images captured throughout the loading process. Overall, the specimens did not undergo severe necking prior to fracture. During early-stage loading, the gauge section deformed uniformly, with only minor cross-sectional contraction observed in the central region at later stages, indicating strain localization, consistent with the eventual fracture location. These observations suggest that the studied ferritic steel exhibits ductile fracture characteristics akin to those of modern advanced high-strength steels (AHSSs). The fracture strain of ferritic steel at 0°, 45°, and 90° are found to be 0.237, 0.220, and 0.212, respectively, which indicates the orientation-related fracture behavior. Consequently, modeling its failure behavior necessitates consideration of stress-state effects.

**Figure 4:**
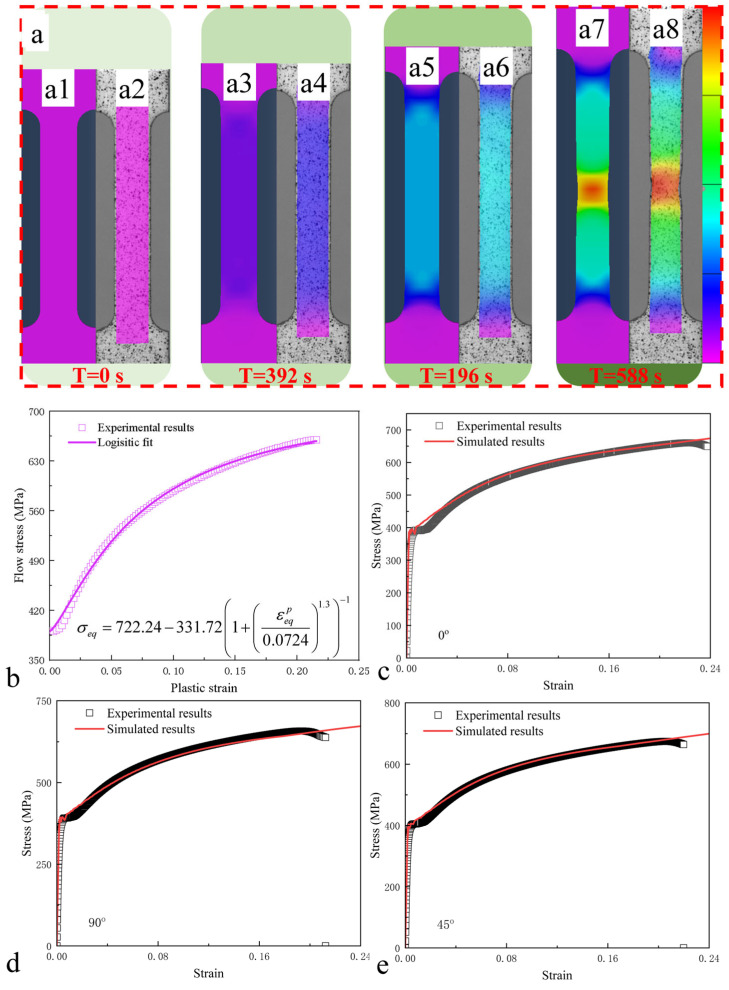
Strain distribution maps of ferritic steel under tension (0°) at different loading stages (**a**), where Figure 4(a1,a3,a5,a7) correspond to numerical simulation results, while Figure 4(a2,a4,a6,a8) represent experimental results. Strain hardening curve (**b**). Comparisons between experimental and numerical results in stress–strain curve at the loading directions of 0° (**c**), 45° (**d**), and 90° (**e**).

Figure 4a presents the strain field distribution of the ferritic steel specimen throughout the deformation process, with Figure 4(a2,a4,a6,a8) displaying the results obtained from DIC experiments. The strain distribution indeed confirms the aforementioned observation that the deformation remains nearly uniform across the gauge section during early loading stages, with strain localization occurring only in later stages until fracture initiation. To characterize the anisotropic plastic behavior of the ferritic steel, this study employs the Hill48 anisotropy criterion, which is widely adopted for engineering metallic materials. Under a general stress state, the Hill48 yield criterion takes the following form:

(1) $$\sigma_{eq}^{2}=F{\left(\sigma_{yy}-\sigma_{zz}\right)}^{2}+G{\left(\sigma_{zz}-\sigma_{xx}\right)}^{2}+H{\left(\sigma_{xx}-\sigma_{yy}\right)}^{2}+2L\sigma_{yz}^{2}+2M\sigma_{xz}^{2}+2N\sigma_{xy}^{2}=1,$$

where $\sigma_{eq}$, *F*, *G*, *H*, *L*, *M*, and *N* are equivalent stress and anisotropic parameters. $\sigma_{xx}$, $\sigma_{yy}$, $\sigma_{zz}$, $\sigma_{yz}$, $\sigma_{xz}$, and $\sigma_{xy}$ are normal stress components and in-plane shear stress components described in the coordinate system of RD-TD-ND. In a 2D situation, the Hill48criterion can be written as:

(2) $$\sigma_{eq}^{2}=F{\left(\sigma_{yy}\right)}^{2}+G{\left(\sigma_{xx}\right)}^{2}+H{\left(\sigma_{xx}-\sigma_{yy}\right)}^{2}+2N\sigma_{xy}^{2}.$$

Then, the anisotropic parameters in Equation (1) can be obtained through the following equations:

(3) $$\begin{array}{l} F=\frac{1}{2}\left(\frac{\sigma_{0}^{2}}{\sigma_{90}^{2}}-1+\frac{\sigma_{0}^{2}}{\sigma_{EB}^{2}}\right)\\ G=\frac{1}{2}\left(1-\frac{\sigma_{0}^{2}}{\sigma_{90}^{2}}+\frac{\sigma_{0}^{2}}{\sigma_{EB}^{2}}\right)\\ H=\frac{1}{2}\left(1+\frac{\sigma_{0}^{2}}{\sigma_{90}^{2}}-\frac{\sigma_{0}^{2}}{\sigma_{EB}^{2}}\right)\\ N=\frac{1}{2}\left(4\frac{\sigma_{0}^{2}}{\sigma_{45}^{2}}-\frac{\sigma_{0}^{2}}{\sigma_{EB}^{2}},\right)\\ L=M=\frac{3}{2} \end{array}$$

where $\sigma_{0}$, $\sigma_{45}$, $\sigma_{90}$, and $\sigma_{EB}$ are the initial yield strength of ferrite steel specimens at different orientations, i.e., 0°, 45°, 90°, and equal biaxial tension, respectively. In order to calibrate the anisotropic parameters, $\sigma_{EB}$ is assumed to be equal to $\frac{\sigma_{0}+2\sigma_{45}+\sigma_{90}}{4}$ [29,30]. The initial yield strength of the material is defined as the stress corresponding to a 0.2% offset strain, from which the yield strengths of ferritic steel with different orientations and the anisotropic parameters based on Hill48 criterion can be obtained as listed in Table 1. It should be emphasized that the anisotropic yield parameters of the material adopted in this work remain constant during plastic flow. This implies that the hardening behavior is described solely by the formula shown in Figure 4b, while the shape of the yield surface remains fixed. In other words, the constitutive modeling framework employed in this study is constructed based on the concept of initial anisotropic yield geometry combined with isotropic hardening.

To accomplish the anisotropic constitutive modeling of the ferritic steel, a continuous description of the material’s strain hardening behavior is required. The tensile flow stress-plastic strain curve of the ferritic steel specimen at an orientation of 0° was fitted using a Logistic function through the Levenberg–Marquardt algorithm, with the fitting results presented in Figure 4b, demonstrating that the Logistic function effectively captures the strain hardening characteristics of the ferritic specimen. To validate the predictive accuracy of the adopted constitutive model for the tensile behavior of ferritic material, numerical simulations of tensile tests for specimens with different orientations were performed by implementing the developed ferritic steel constitutive relationship into ABAQUS via VUMAT, incorporating the return mapping algorithm combined with the associated flow rule.

The yield criterion based on the Hill48 function can be written as:

(4) $$\begin{array}{l} F(\sigma_{ij})=f(\sigma_{ij})-\sigma_{eq}\\ \;\;\;\;\;\;\;={\left(F{\left(\sigma_{yy}-\sigma_{zz}\right)}^{2}+G{\left(\sigma_{zz}-\sigma_{xx}\right)}^{2}+H{\left(\sigma_{xx}-\sigma_{yy}\right)}^{2}+2L\sigma_{yz}^{2}+2M\sigma_{xz}^{2}+2N\sigma_{xy}^{2}\right)}^{1/2}-\sigma_{eq.} \end{array}$$

Then, the flow direction can be obtained based on the assumption of the associated flow rule:

(5) $$\frac{\partial F(\sigma_{ij})}{\partial \sigma_{ij}}=\frac{\partial f(\sigma_{ij})}{\partial \sigma_{ij}}={\left[\begin{array}{c} \frac{H(\sigma_{xx}-\sigma_{yy})-G(\sigma_{zz}-\sigma_{xx})}{\sigma_{eq}}\\ \frac{F(\sigma_{yy}-\sigma_{zz})-H(\sigma_{xx}-\sigma_{yy})}{\sigma_{eq}}\\ \frac{G(\sigma_{zz}-\sigma_{xx})-F(\sigma_{yy}-\sigma_{zz})}{\sigma_{eq}}\\ \frac{2N}{\sigma_{eq}}\\ \frac{2L}{\sigma_{eq}}\\ \frac{2M}{\sigma_{eq}} \end{array}\right]}^{T}.$$

For homogeneous function, the quantitative relation between increments of equivalent plastic strain $d\varepsilon_{eq}^{p}$ and the increment of plastic multiplier dλ can be derived with consideration of the principle of plastic work equivalence:

(6) $$d\lambda =d\varepsilon_{eq}^{p}.$$

Furthermore, the specific form of dλ can be obtained as:

(7) $$d\lambda =\frac{\frac{\partial f(\sigma_{ij})}{\partial \sigma_{ij}}:C_{ijkl}:d\varepsilon \varepsilon_{kl}}{\frac{d\sigma_{eq}^{p}(\varepsilon_{eq}^{p})}{d\varepsilon_{eq}^{p}}+\frac{\partial f(\sigma_{ij})}{\partial \sigma_{ij}}:C_{ijkl}:\frac{\partial f(\sigma_{kl})}{\partial \sigma_{kl}}},$$

where $C_{ijkl}$ is the elastic modulus tensor and the hardening modulus $\frac{d\sigma_{eq}^{p}(\varepsilon_{eq}^{p})}{d\varepsilon_{eq}^{p}}$ can be expressed as:

(8) $$\frac{d\sigma_{eq}^{p}(\varepsilon_{eq}^{p})}{d\varepsilon_{eq}^{p}}=6003{\left(13.82\varepsilon_{eq}^{p}\right)}^{0.31}{\left(1+{\left(\frac{\varepsilon_{eq}^{p}}{0.0724}\right)}^{1.3}\right)}^{-2}.$$

Therefore, given the explicit return mapping algorithm:

(9) $$\begin{array}{l} \varepsilon_{ij,n+1}=\varepsilon_{ij,n}+\Delta \varepsilon_{ij}\\ \varepsilon_{ij,n+1}^{p}=\varepsilon_{n}^{p}+\Delta \lambda_{n}{\left.\frac{\partial f(\sigma_{ij})}{\partial \sigma_{ij}}\right|}_{n}\\ \varepsilon_{eq,n+1}^{p}=\varepsilon_{eq,n}^{p}+\Delta \lambda_{n}{\left.\frac{d\sigma_{eq}^{p}(\varepsilon_{eq}^{p})}{d\varepsilon_{eq}^{p}}\right|}_{n}\\ \sigma_{ij,n+1}=C_{ijkl}:(\varepsilon_{kl}-\varepsilon_{kl,n+1.}^{p}) \end{array}$$

Therefore, the built constitutive model can be carried out in ABAQUS to simulate the tension behavior of ferritic steel specimens at various orientations. Figure 4c–e display the comparisons between experimental and numerical results in the stress–strain curve at the loading directions of 0°, 45°, and 90°, respectively. Results show that the experimental data agrees well with the numerical results which indicates the reliability of constitutive relations. In addition, Figure 4a displays the strain distribution contours of the ferritic steel under tensile loading (0° orientation) at different loading stages, where Figure 4(a1,a3,a5,a7) correspond to numerical simulation results, while Figure 4(a2,a4,a6), and 4(a8) represent experimental results. It can be seen that the simulations based on the built anisotropic constitutive model reproduce the experimental results. This phenomenon further corroborates that the established material constitutive model can effectively predict the mechanical behavior of the material. In addition, at the onset of necking (Figure 4a), the ultimate tensile strength is approximately 657 MPa. At this stage, the FEM results show a maximum strain of 0.20566 in the necking zone, while the DIC results give a maximum strain of 0.19708, corresponding to an error of approximately 4.4%. Therefore, it can be concluded that the established FEM model can effectively predict the anisotropic mechanical behavior of the ferritic steel.

### 3.3. Fracture Behaviors

**Figure 5:**
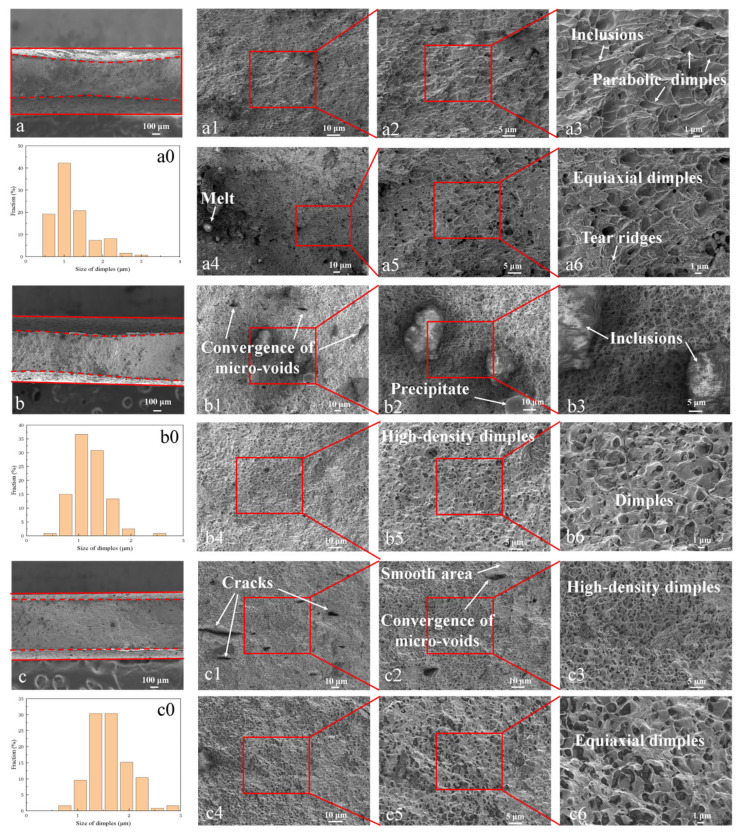
SEM images of fracture morphology of deformed specimens at an orientation of 0° (**a**), 45° (**b**), and 90° (**c**).

Figure 5 presents SEM images of fracture morphologies for ferritic steel specimens with different orientations after tensile failure. Overall, all specimens exhibit ductile fracture characteristics regardless of orientation. For the 0° orientation, the macroscopic fracture surface appears flat with noticeable thickness reduction at the central region, while the lateral edges show less pronounced contraction, resulting in a “thick ends-thin middle” cross-sectional profile as clearly visible in Figure 5a. The red dashed lines in Figure 5a–c indicate the post-fracture cross-sectional boundaries, whereas the solid red lines represent the initial specimen geometry. The fracture surface predominantly consists of dimples, including both parabolic and equiaxed types, suggesting a mixed tensile-shear fracture mechanism. For the 0° specimen, the average dimple size on the fracture surface is approximately 1.18 μm, with a maximum measured size of 3.14 μm and a minimum of 0.46 μm within the statistical range. The detailed size distribution is presented in Figure 5(a0). The absence of second-phase particles at dimple bottoms indicates that dimple nucleation primarily occurred at grain boundaries. Additional features include localized melting and scattered inclusions (identified as precipitates based on their surface distribution rather than dimple-bottom locations).

The 45° orientation specimen displays similar overall contraction but with distinct anisotropic deformation characteristics, exhibiting an asymmetric “thick-thin” cross-section instead of symmetrical necking. Microscopic examination reveals microvoid coalescence, second-phase particles (~25 μm), smooth-surfaced precipitates (~20 μm), and high-density dimples of both parabolic and equiaxed types. The average dimple size is approximately 1.21 μm, with a maximum measured size of 2.46 μm and a minimum of 0.52 μm within the statistical range. The detailed size distribution is presented in Figure 5(b0).

For the 90° orientation, the fracture surface demonstrates uniform thickness reduction with a flat morphology, featuring microcrack coalescence accompanied by smooth cleavage-like planes (attributed to rapid tearing during void linkage) alongside predominant equiaxed dimples, indicating typical tensile-dominated fracture mechanisms. For the 90° specimen, the average dimple size on the fracture surface is approximately 1.6 μm, with a maximum measured size of 2.80 μm and a minimum of 0.71 μm within the statistical range. The detailed size distribution is presented in Figure 5(c0).

It is worth noting that the observed orientation-dependent fracture behavior may also be rationalized from the perspective of local stress triaxiality evolution during post-necking deformation. The strong α-fiber texture (<110>||RD, ≈59.8%) gives rise to anisotropic plastic flow, which in turn affects the development of the triaxial stress state within the necking region. For the 0° and 90° orientations, the texture promotes a more diffuse neck with lower local stress triaxiality, favoring microvoid coalescence and leading to a predominantly equiaxed dimple morphology and higher fracture strain. In contrast, for the 45° orientation, the texture induces a sharper strain localization and a higher local stress triaxiality in the necked zone, which promotes earlier void nucleation and coalescence under a mixed tensile-shear stress state, resulting in a lower fracture strain (0.220) and a mixture of parabolic and equiaxed dimples.

In addition to the crystallographic characterization, it is worth discussing the possible nature of the precipitates and inclusions observed on the fracture surfaces (Figure 5), as they are potential void nucleation sites during tensile deformation. In ferritic steels, two common types of secondary phases are typically encountered: Nb-rich carbides (e.g., NbC or (Nb,Ti)_2_C) and Fe_2_Nb-type or Fe_2_(Nb,W)-type Laves phases [24]. It has been reported that Nb-bearing carbides and Laves phases can co-exist and even undergo mutual transformation during thermal processing. Moreover, coarser Cr-rich carbides are known to act as preferential damage nucleation sites [14,31], promoting void nucleation and coalescence under tensile loading. In the present study, the observed inclusions and precipitates vary in size and surface morphology. Although their precise phase identification would require further analytical techniques such as energy-dispersive X-ray spectroscopy (EDS) or transmission electron microscopy (TEM), it is plausible that the finer precipitates correspond to Nb-rich carbides or Laves phases, whereas the larger inclusions may be Cr-rich carbides or complex oxide-based non-metallic inclusions. These second-phase particles, regardless of their specific type, can act as preferential sites for void nucleation by either particle decohesion from the ferrite matrix or particle cracking, thereby contributing to the observed ductile fracture characterized by dimple-dominated morphologies.

### 3.4. Limitations and Future Work

The present work comprehensively investigates and analyzes the mechanical behavior, deformation characteristics, constitutive modeling, and failure mechanisms of ferritic steel with different crystallographic orientations under tensile loading. The Hill48 criterion is found to be capable of reasonably describing the tensile mechanical properties of ferritic steel. However, the current study has the following limitations: (1) the range of investigated orientations requires expansion to cover a broader angular spectrum; (2) the microstructural evolution during deformation has not been addressed; (3) phase identification of precipitates involved in the fracture mechanisms was not performed; (4) establishing an anisotropic fracture criterion for ferritic steel should be a key focus of future research; (5) the constitutive model adopted in this paper is based on the associated flow rule assumption. This assumption may become invalid when the principal directions of stress and strain are not aligned during plastic flow, which necessitates the additional consideration of an anisotropic plastic potential function to accurately describe the deformation characteristics of the material.

In view of the above limitations, the following research directions are suggested for future work: quasi-in situ or in situ tests should be conducted to investigate the microstructural evolution of ferritic steel during deformation, which would not only enable quantitative analysis of microstructural changes but also help establish a link between microstructure and macroscopic mechanical properties. Furthermore, mechanical tests on ferritic steel under a wider range of testing conditions (e.g., multiaxial stress states) should be carried out to establish a yield criterion that accurately describes the material’s mechanical behavior in the full stress space. Finally, in situ TEM techniques should be employed to analyze the formation mechanisms of precipitates in ferritic steel and their corresponding failure mechanisms.

## 4. Conclusions and Remarks

This study comprehensively investigates the orientation-dependent tensile mechanical properties of ferritic steel. The results demonstrate significant orientation sensitivity in the inelastic behavior, including plastic response and fracture characteristics, primarily attributable to rolling-induced texture. Specifically, yield strengths are 391 MPa (0° and 90°) and 405 MPa (45°), while fracture strains are 0.237, 0.220 and 0.212 for the 0°, 45° and 90° orientations, respectively. EBSD characterization reveals an average grain size of 8.01 μm and a dominant α-fiber texture ({110}//RD, 59.8%), which is the primary source of the anisotropic mechanical response. The Hill48 yield criteria, calibrated using the orientation-dependent yield stresses, accurately predict the tensile behavior of all three orientations when implemented into a finite element framework, as validated by digital image correlation measurements. All specimens fail in a ductile manner with dimple-dominated fracture surfaces, and no brittle cleavage is observed. From a practical perspective, the validated constitutive model provides a reliable tool for forming the process design of ferritic steel sheets. To minimize premature fracture and optimize deep-drawing performance, the rolling direction should be aligned with the 0° or 90° orientation (which offer higher fracture strain) rather than the 45° direction.

## Figures and Tables

**Table 1:** Orientation-related yield strength (dimension in MPa) and anisotropic parameters.

Orientation				Anisotropic Parameters					
$\sigma_{0}$	$\sigma_{45}$	$\sigma_{90}$	$\sigma_{EB}$	*F*	*G*	*H*	*N*	*M*	*L*
391	405	391	398	0.483	0.483	0.517	1.382	1.5	1.5

## Data Availability

The original contributions presented in this study are included in the article. Further inquiries can be directed to the corresponding authors.
